# Prevention of unintentional injuries in children under five years

**DOI:** 10.1186/s12887-021-02517-2

**Published:** 2021-09-08

**Authors:** Sophie Jullien

**Affiliations:** Barcelona Institute for Global Health, University of Barcelona, Barcelona, Spain

**Keywords:** Prevention, Safety, Injuries, Traffic accident, Drowning, Poisoning, Burning, Accidental falls

## Abstract

We looked at existing recommendations for preventing unintentional injuries in children under five years of age, and we attempted to identify the main sources used as evidence for formulating these recommendations.

We conducted a literature search up to the 18th October 2019 by using key terms and manual search in selected sources. We summarized the recommendations and source of the evidence in tables for each of five areas of unintentional injuries: road traffic injuries, drowning, poisoning, thermal injuries, falls.

In 2008, the World Health Organization (WHO) published a comprehensive report with strategies for child injury prevention for the European region. More recently, the WHO published several guidance documents focused on one area such as drowning, usually with a global focus. The PrevInfad workgroup (Spanish Association of Primary Care Pediatrics) updated their document on road safety in April 2019, providing recommendations and a summary of the existing evidence. Preventive strategies for injuries in childhood are mainly based on surveillance data and the identification of risk factors. The key strategies for preventing unintentional injuries are a combination of environmental and behaviour modification, that can be achieved through engineering, enforcement and education. Consequently, for this kind of strategies, it is important to evaluate the effectiveness of both the intervention itself, and the way the intervention is advised to parents and caretakers so that there is good compliance of the recommendation.

## Background

### Introduction

The World Health Organization (WHO) European Region is developing a new pocket book for primary health care for children and adolescents in Europe. This article is part of a series of reviews, which aim to summarize the existing recommendations and the most recent evidence on preventive interventions applied to children under five years of age to inform the WHO editorial group to make recommendations for health promotion in primary health care. In this article, we looked at existing recommendations for preventing unintentional injuries in children under five years of age, and we attempted to identify the main sources used as evidence for formulating these recommendations.

### What are unintentional injuries?

An injury is ‘the physical damage that occurs when the human body is suddenly subjected to amounts of energy that exceed the physiological threshold or is deprived of vital elements such as oxygen’ [1]. ‘The energy can be mechanical, thermal, chemical or radiant’. The main causes of unintentional injuries are road traffic injuries, drowning, poisoning, thermal injuries and falls.

### Context

Unintentional injuries cause death and long-term disabilities in many children in the WHO European Region. Already in 1958, the WHO European Region Office recognized injuries as ‘the leading cause of childhood death in Europe’ [1]. Furthermore, the number of deaths is only the tip of the injury pyramid. For each child who died from unintentional injuries, many more are admitted to the hospital, and even more attend emergency departments [2]. Among children under five years of age, most of the unintentional injuries happen at home. The main causes of death in this age group are choking, suffocation or strangulation (49%), drowning (22%), falls (8%), and smoke, fire and flames (8%).The main causes of hospital admissions are falls (52%), being struck by objects (22%), poisoning (13%), and heat or hot substances (6%), as per data from Public Health England in 2014 [2].

However, injuries are an important preventable cause of premature death. In 2015, the Royal Society for the Prevention of Accidents in England estimated that unintentional injuries constitute between 60 to 65% of preventable deaths among children under five years of age, and more than 85% of preventable hospital admissions among children under nine years of age [2].

Therefore, there is a huge potential for prevention. ‘The experience accumulated by several European countries shows that sustained and systematic approaches that address the underlying causes of injuries, such as their socioeconomic and environmental determinants, can make all countries in the Region among the safest in the world’ [1].

## Key questions

1. **What are the main recommendations for preventing unintentional injuries in children under five years of age?**
2. **What are the main sources used as evidence for formulating the above recommendations?**

## Methods

The methods of all the articles of this Supplement, including the selection process of preventive interventions, search methods, data collection and data synthesis, and limitations, are summarized in the article [Jullien S, Huss G, Weige R. Supporting recommendations for childhood preventive interventions for primary health care: elaboration of evidence synthesis and lessons learnt. BMC Pediatrics. 2021. 10.1186/s12887-021-02638-8]. Prevention of unintentional injuries in children is a broad area comprising preventive measures for traffic injuries, drowning, poisoning, burns, falls, etc. Summarising all the existing recommendations and conducting a search to identify all the systematic reviews and other relevant studies for each of the areas included in prevention of unintentional injuries in childhood is beyond the scope of this document. Therefore, we used a different approach for this topic compared to the rest of the preventive interventions we have summarized in the other articles of this supplement (Jullien S, Huss G, Weige R. Supporting recommendations for childhood preventive interventions for primary health care: elaboration of evidence synthesis and lessons learnt. BMC Pediatrics. 2021. 10.1186/s12887-021-02638-8). We have selected five areas of unintentional injuries that we estimated to be the most relevant as for prevalence and severity, following the classification usually used by the WHO: 1) road traffic injuries; 2) drowning; 3) poisoning; 4) thermal injuries; 5) falls. For each of the selected area, we report existing recommendations given by selected guidelines or institutions and provide illustrative systematic reviews and other relevant studies that the guidelines used in formulating their recommendations.

We conducted the search up to the 18th of October 2019, by manual search and by using the search terms: ‘road traffic accident’ OR ‘unintentional injuries’ OR ‘pedestrian’ OR ‘child restraint device’ OR ‘helmet’ OR ‘drowning’ OR ‘poisoning’ OR ‘intoxication’ OR ‘burn’ OR ‘thermal’ OR ‘scald’ OR ‘fall’ within child health OR ‘accident prevention’. We searched the same sources as for the other articles of this supplement, those are: the WHO, the US Preventive Services Task Force (USPSTF), PrevInfad, the Royal College of Paediatrics and Child Health (RCPCH), the Centers of Disease Control and Prevention (CDC), the National Institute for Health and Care Excellence (NICE), and the Cochrane library. We contacted field experts for additional references such as reports from meetings. For the sources of the evidence, we report systematic reviews and trials we deemed relevant, cited as references for supporting the identified strategies, as well as those identified by directly searching in the Cochrane library. In most cases, the documents reporting the strategies and recommendations present a broader and more extensive list of references; some documents summarised the main findings. The list we provide is therefore not exhaustive but attempts to be rigorous and methodical.

We summarise the recommendations and source of the evidence in tables for each of the five selected areas.

## Findings: existing recommendations and source of the evidence

Evidence indicates that a range of different approaches can prevent or reduce the impact of injuries, commonly referred as the 3 Es: 1) Engineering, for modifying products and modifying the environment to make home safer; 2) Enforcement, through laws, regulations and standards; and 3) Education and skills development, including emergency health care [1–3].

Randomized controlled trials that compare injuries or deaths between children exposed and unexposed to a preventive strategy or measure are most of the times not feasible and unethical. Most of the data come from surveillance data, and retrospective analysis, that identify risk factors and risk patterns, which assist in the development of strategies to reduce the risk. Consequently, due to the nature of the topic and the lack of supportive evidence, some recommendations are characterized as ‘good practice recommendations.’ Those are ‘overarching principles derived not from scientific evidence but from common sense and established international agreements on ethics and human rights’ [4].

In this document, we summarize recommendations and sources of evidence by type of injury. However, the evidence supporting approaches that work for prevention goes beyond this. On the one hand there is a need to understand which preventive interventions work for preventing unintentional injuries, for example whether installing fencing around a pool will prevent drowning. On the other hand, there is a need to understand by which means it is most effective to put the preventive intervention into practice. That is, how to make families to install fencing around their pool. The PrevInfad and the RCPCH groups assessed the evidence around these aspects on a structured way in their respective documents on road safety and book chapter [2, 5].

### Road traffic injuries

In 2008, the WHO estimated that road traffic injuries were the leading cause of fatal injuries in children and a leading cause of traumatic head and extremity injuries with consequent long-term disability throughout the WHO European Region [1]. The Sustainable Development Goals adopted by the United Nations in 2015 included two specific targets related with road safety, which confirms safety as ‘an essential element of the health and development agenda’ [6].

Several publications on road traffic safety are available from the WHO, ‘which have been developed jointly by multiple partner agencies of the UN Road Safety Collaboration, or in some cases by individual partner agencies’ [7]. Other resources can be found under the WHO ‘Violence and Injury Prevention’ section [8]. We have selected the manuals that provide information and recommendations on child restraint systems, helmets for riders and passengers, and pedestrian safety. The WHO European report on child injury is a comprehensive report that is described by the authors as ‘a companion to the World report on child injury prevention and focuses on the European dimension of the problem’ [1, 9]. In this report, the WHO classified their recommendations and key strategies to reduce motor vehicle injuries in children up to 19 years as either effective, promising, insufficient evidence, ineffective, or harmful. The strategies cited as effective were: zero-tolerance alcohol laws, laws on minimum legal drinking age, lower blood alcohol concentration levels, mass-media publicity, child safety seats, booster seats, motorcycle helmets, bicycle helmets, graduated driver licensing systems, and rear seating position. There was insufficient evidence for the strategies on education-only programmes for child-seat use, designates-driver programmes, increasing visibility of vulnerable road users, and school-based instruction programmes for drinking and driving. School-based driver education was considered an ineffective strategy, and airbags and children, and early licensure for novice teenage drivers were classified as harmful strategies [1].

PrevInfad has recently updated their recommendation (April 2019) and provided a comprehensive document summarising the existing evidence around measures for preventing traffic injuries in children [5]. The PrevInfad Group recommends that ‘primary care professionals offer counselling on the use of child restraint systems and the use of helmet in bicycles and motorbikes, in the well child visits and in other favourable situations such as care in case of traffic injury of any seriousness’ (B recommendation) and that ‘the Pediatrics’ professional, when performing community work proper to primary care, take part and promote this kind of educational campaigns in his/her setting’ (B recommendation). The group concludes that ‘there is insufficient evidence on the effectiveness of brief counselling in the medical office on road safety education, child and adolescent safety as drivers, child as motorbike passenger and school transport’ (I statement) [5].

The USPSTF have decided not to update their 2007 recommendations. ‘Based on the currently available evidence, the USPSTF believes the potential impact of a new USPSTF recommendation on counselling for proper use of motor vehicle occupant restraints and avoidance of alcohol use to prevent injury is limited’ [10]. Therefore, the USPSTF refers clinicians to the guidance from the CDC.

In 2010, NICE published three guidance documents on how to prevent unintentional injuries in children under 15 years of age. One of them addressed unintentional injuries on the road [11].

EuroSafe, the European Association for Injury Prevention and Safety Promotion, was created with the mission ‘to prevent home and leisure accidents by working in partnership with industry, governments, research institutes and health and safety practitioners to help reduce the greatest risks’ [12]. In 2006, they developed Good Practice Guidance for Child Safety, addressing prevention of unintentional child injuries and promotion of safety [13]. This guide is focused on Europe and addresses all the selected areas we present in this summary, providing evidence-based recommendations and methodology.

Aware of the importance of the topic and the need of updating evidence-based recommendations, the WHO has very recently made a call ‘to review literature on new evidence on selected road traffic injury risk factors and interventions’ [14]. The WHO is seeking to assemble new evidence about a series of risk factors and interventions measures that include not wearing a helmet, not wearing a seat-belt, and a child restraint and pedestrian safety.

We summarize in Table 1 the recommendations and sources of the evidence for the prevention of traffic road injuries, focusing on child restraint system, the use of helmets, and safety education of pedestrians.

**Table 1:** Recommendations and sources of the evidence for preventing traffic road injuries

Recommendations	Guidelines and/or institutions recommending the intervention	Source of the evidence
**Child restraint system** - Correct use of the child restraint system - Rear-facing (as long as possible) - Position in rear centre seat	- WHO 2008; European region [1] - WHO 2018; global report [6] - WHO 2009 [15] - PrevInfad 2019 (high degree of certainty that net profit is important) [5] - RCPCH 2019 [2] - CDC 2019 [16] - EuroSafe 2006 [13]	**Use of child restraint system:** - Arbogast 2009; cohort study [17] - Zaloshnja 2007; cohort study [18] - Elliott 2006; cohort study [19] - Sauber-Schatz 2015; cohort study [20] **Rear-facing:** - MAPFRE 2011; Spanish summary report [21] - McMurry 2018; cohort study [22] - Jakobsson 2005, 40-year Swedish cohort [23] - Gloyns 2008; European study commissioned by ANEC [24] **Rear seat:** - Lennon2008; cohort study [25] - Kallan 2008; cohort study [26] **Promotion of interventions:** - Ehiri 2006; Cochrane systematic review [27]
**Helmet** - Use of helmet for all children, as riders and passengers in bikes and motorcycles	- WHO 2008; European region [1] - WHO 2018, global report [6] - WHO 2006 [28] - PrevInfad 2019 [5] - CDC 2019 [16]. CDC provides a set of recommendations on how to choose a helmet, how to wear it correctly, how to take care of it, and when to replace it. - USCPSTF 2013 [29]. Strong evidence for recommendation on universal motorcycle helmet laws. - EuroSafe 2006 [13]	- Owen 2012; Cochrane systematic review [30] - Liu 2008; Cochrane systematic review [31] - Macphersen 2008; Cochrane systematic review [32] - Thompson 1999; Cochrane systematic review [33] - USCPSTF 2013; review of the literature [29]
**Safety education of pedestrians** - Pedestrians can increase their visibility at night - Cross the street at a designated crosswalk or intersection - Walk on a sidewalk or path; if not available, walk on the shoulder and facing traffic	- WHO 2008; European region [1] - WHO 2018, global report [6] - WHO 2013 [34] - PrevInfad 2019 [5] - RCPCH 2019 [2] - CDC 2019 [16] - NICE 2010a [35] - EuroSafe 2006 [13]	- Kwan 2009 [36] - Duperrex 2002; Cochrane systematic review [37, 38] - Turner 2004; systematic review [39] - Bunn 2003; Cochrane systematic review [40]

***Abbreviations*:***CDC* Centers for Disease Control and Prevention, *NICE* National Institute for Health and Care Excellence, *PrevInfad* PrevInfad workgroup from the Spanish Association of Primary Care Pediatrics, *RCPCH* Royal College of Paediatrics and Child Health, *USCPSTF* US Community Preventive Services Task Force, *WHO* World Health Organization

### Drowning

Drowning is ‘the process of experiencing respiratory impairment from submersion/immersion in liquid’ [1]. In 2008, the WHO estimated that drowning was the second leading cause of injury deaths among children in the WHO European region, and the leading cause in some countries [1]. Among children between one and 4 y of age, drowning represents the first cause of deaths caused by unintentional injuries. In addition, children who survive drowning may present severe long-term disabilities. There are inequalities among European countries and between socioeconomic statuses. It was estimated that ‘the poorest people are up to 11 times more vulnerable to drowning than the rich’, and that ‘if rates in all countries matches those of the countries with the lowest rates, 9 out of 10 deaths could be averted’ [1].

The 2008 WHO European report on child injury includes strategies for the prevention of drowning [1]. More recently, the WHO published an implementation guide dedicated only to the prevention of drowning, for a global audience – and not focused to the European context [41]. This latter guideline presents six selected interventions for preventing drowning (see Table 2) and four implementation strategies that underpin the interventions: strengthen public awareness of drowning through strategic communications, promote multisectorial collaboration, develop a national water safety plan, and advance drowning prevention through data collection and well-designed studies. The authors selected these interventions and strategies ‘as the best evidence-based ways to prevent drowning’ and specified that ‘many of them are more commonly used in high-income countries’ [41].

**Table 2:** Recommendations and sources of the evidence for preventing drowning

Recommendations	Guidelines and/or institutions recommending the intervention	Source of the evidence
**Installation of a four-sided isolation pool fencing** With self–closing and self–latching gates, around backyard swimming pools. Pool fences should completely separate the house and play area from the pool.	- WHO 2008; European region [1] - WHO 2017; global [41] - PrevInfad 2011 [42] - CDC 2019 [16] - EuroSafe 2006 [13]	- Thompson 1998; Cochrane systematic review [43] - Neto 2008, review [44]
**Provide safe places away from water for pre-school children** **Removing or covering water hazards**	- WHO 2008; European region [1] - WHO 2017; global [41] - RCPCH 2019 [2]	(*See original documents*)
**Use of life jackets and personal flotation devices** Make sure kids wear life jackets in and around natural bodies of water, such as lakes or the ocean, even if they know how to swim. Life jackets can be used in and around pools for weaker swimmers too.	- WHO 2008; European region [1] - CDC 2019 [16]	(*See original documents*)
**Set and enforce safe boating, shipping and ferry regulations**	- WHO 2017; global [41] - EuroSafe 2006 [13]	(*See original documents*)
**Swimming** Everyone should know the basics of swimming (floating, moving through the water)	- WHO 2008; European region [1] - WHO 2017; global [41] - CDC 2019 [16] - NICE 2010b [35] - EuroSafe 2006 [13]	(*See original documents*)
**Close supervision at all times** When kids are in or near water (including bathtubs). Because drowning happens quickly and quietly, adults watching kids in or near water should avoid distracting activities like playing cards, reading books, talking on the phone, and using alcohol or drugs.	- RCPCH 2019 [2] - CDC 2019 [16] - NICE 2010b [35]	- Kendrick 2012; Cochrane systematic review [45] - Epidemiological surveillance of drowning in France, 2015 [46]
**Provide immediate resuscitation** - Perform immediate cardiopulmonary resuscitation to drowning victims - Train and employ lifeguards at beaches and public swimming pools	- WHO 2008; European region [1] - WHO 2017; global [41] - RCPCH 2019 [2] - NICE 2010b [35] - EuroSafe 2006 [13]	(*See original documents*)

***Abbreviations*:***CDC* Centers for Disease Control and Prevention, *NICE* National Institute for Health and Care Excellence, *PrevInfad* PrevInfad workgroup from the Spanish Association of Primary Care Pediatrics, *RCPCH* Royal College of Paediatrics and Child Health, *WHO* World Health Organization

PrevInfad recommendations date from 2011; the Group is currently updating this chapter [42]. Under the section on ‘home safety’, they assessed the effectiveness of advice on different areas, for example, how effective it is to give advice to install pool fencing for the increasing the number of families who install them [42].

One of the three NICE guides published in 2010 recommends some actions to be taken for preventing drowning in children [35].

We identified one Cochrane systematic review, published in 1998, that assessed the pool fencing for preventing drowning in children. The rest of the evidence comes mainly from national, state or provincial health data collection systems, national databases on drowning information, and surveys. The 2017 global WHO guide provides a broad bibliography.

We summarize in Table 2 the recommendations and sources of the evidence for prevention of drowning – or child water safety.

### Poisoning

Poisoning refers to ‘an injury sustained due to exposure to a substance that causes cellular injury or death’ [1]. The exposure to the substance can be by ingestion, inhalation, injection or absorption. Although chronic exposition also represents concern for health, we herein refer to poisoning due to acute exposure.

Poisoning is the third leading cause of unintentional injury death in the European Region, as estimated by the WHO in 2008 [1]. Non-fatal poisonings are even more frequent and constitute an important cause of emergency admissions and long-lasting disability. The poorest country in the European Region concentrate 90% of the deaths associated to poisoning, and the WHO estimated that ‘if all countries in the region had the same rate as the country with the lowest, seven out of ten deaths could be averted’ [1].

The 2008 WHO European report on child injury classified their recommendations and key strategies to prevent poisoning as effective, promising, insufficient evidence, or ineffective [1]. Four key strategies were classified as effective: removing the toxic agent, passing and enforcing legislation requiring child-resistant packaging of medicines and poisons, packaging drugs in non-lethal quantities, and establishing poison control centres. Locking away medicines and other toxic substances was considered promising. Four strategies presented insufficient evidence: teaching children to avoid poisonous substances, reducing the attractiveness of medications and poisonous products, and providing home safety education and safety equipment; and one was considered ineffective: clearly labelling toxic products. Once more, these key strategies are a combination of environmental and behaviour modification, that can be achieved through engineering, legislation and education.

Other sources and information can be found in the WHO website [47]. The IPCS INTOX project was launched in the UK in 1988, as a result of collaboration among several international institutions, ‘to promote chemical safety through the establishment and strengthening of poisons centres’ [48].

As mentioned for drowning, PrevInfad recommendations date from 2011 [42]. Under the section on ‘home safety’, they assess whether giving advice on safe storage of medicines and poisons leads families to store these products safely, and results in a decrease in the number of poisonings.

A Cochrane systematic review determined the effect of modifications to the home environment on the reduction of injuries due to environmental hazards [49]. The review authors included safety knowledge, possession, compliance with and use of safety equipment, and disaggregated data for children. We therefore report this review as a source of evidence for the corresponding interventions. Another Cochrane systematic review assessed the effects of parenting interventions for preventing unintentional injury in children (≤18 years) for increasing possession and use of safety equipment and safety practices by parents [50].

We summarize in Table 3 the recommendations and sources of the evidence for prevention of poisoning in children.

**Table 3:** Recommendations and sources of the evidence for preventing poisoning

Recommendations	Guidelines and/or institutions recommending the intervention	Source of the evidence
**Remove the toxic agent**	- WHO 2008; European region [1] - RCPCH 2019 [2]	- Flanagan 2005; cohort study [51] - Roberts 2003; cohort study [52]
**Packaging** - Pass and enforce legislation requiring child-resistant packaging of medicines and poisons - Packaging drugs in non-lethal quantities	- WHO 2008; European region [1] - EuroSafe 2006 [13]	- Flanagan 2005; cohort study [51] - European laws and regulations 2008 [53]
**Poison control centres** - Establishing poison control centres - Having a poison control centre sticker available - Know the nationwide poison control centre phone number	- WHO 2008; European region [1] - PrevInfad 2011 [42] - CDC 2019 [16] - EuroSafe 2006 [13]	- IPCS INTOX Programme [48] - WHO resources for poisons centre [54] - Kendrick 2013; Cochrane systematic review [50] - Kendrick 2012; Cochrane systematic review [45] - Bateman 2002; cohort study [55]
**Secure storage** Lock away medicines and other toxic substances	- WHO 2008; European region [1] - PrevInfad 2011 [42] - RCPCH 2019 [2] - CDC 2019 [16] - EuroSafe 2006 [13]	- Kendrick 2013; Cochrane systematic review [50] - Kendrick 2012; Cochrane systematic review [45] - Turner 2011; Cochrane systematic review [49]
Remove or regulate availability of toxic substances that are easily mistaken for edible items	- WHO 2008; European region [1]	(*See original documents*)
Teach children to avoid poisonous substances	- WHO 2008; European region [1]	(*See original documents*)
Attractiveness of medications and poisons - Reduce the attractiveness of medications and poisonous products - Do not call medicine ‘candy’	- WHO 2008; European region [1] - CDC 2019 [16]	(*See original documents*)
Provide home safety education and safety equipment	- WHO 2008; European region [1]	- Kendrick 2013; Cochrane systematic review [50] - Turner 2011; Cochrane systematic review [49]
**Safe use of syrup of ipecac** Routine administration of ipecac at the site of ingestion or in the emergency department should be avoided	- PrevInfad 2011 [42]	- Kendrick 2012; Cochrane systematic review [45] - Höjer 2013; systematic review [56]

***Abbreviations*:***CDC* Centers for Disease Control and Prevention, *PrevInfad* PrevInfad workgroup from the Spanish Association of Primary Care Pediatrics, *RCPCH* Royal College of Paediatrics and Child Health, *WHO* World Health Organization

### Thermal injuries

A thermal injury is ‘an injury to the skin and other organic tissue caused by thermal trauma’ [1]. Thermal injuries include house fires, contact burns (from hot solids) and scalds (from hot liquids). Fires are a leading cause of death in children, while contact burns and scalds can cause considerable disfigurement and long-term disability. Children under five years of age are at the highest risk for hospital admissions due to thermal injuries. Once more, inequalities exist and if all countries matched the lowest rate in the European region, 90% of deaths could be prevented [1].

The 2008 WHO European report on child injury give recommendations for preventing thermal injuries, with a focus to the European region. The same year, the WHO published a global report known as the ‘Burn plan’ [57]. In 2011, the WHO published a document dedicated to burn prevention, which compiles ‘successful burn prevention strategies from around the world, and from a wide spectrum of economic situations’ [3]. Smoke alarms, hot water heater temperature regulation, flame-retardant sleepwear, electrical safety, sprinklers, child-resistant lighters, fire-safe cigarettes, and banning the manufacture and sale of fireworks are strategies with proven or promising evidence of effectiveness. This was assessed based on several types of data including ‘laboratory evidence (where required), data from individual epidemiological studies or controlled trials, and, for some, evidence of sustained decreases in population-based burn rates over 10 or more years in wide areas or entire countries’ [3]. The references of such data can be found in the original document. The review authors specified that these examples are from high-income countries. The document also includes burn prevention strategies that are promising. Nevertheless, they are mainly focused to low- and middle-income countries (for example safer stoves and lamps, or acid-throwing) and we therefore do not include them in the present summary.

A 2010 NICE guidance document on prevention strategies for unintentional strategies recommend some actions to be taken for preventing thermal injuries in children [35].

The Cochrane systematic review that looked at the effect of modifications to the home environment on the reduction of injuries due to environmental hazards included some interventions related with thermal injuries [49]. Another Cochrane review assessed ‘the effects of community-based interventions defined as coordinated, multi-strategy initiatives, for reducing burns and scalds in children aged 14 years and under’ [58].

We summarize in Table 4 the recommendations and sources of the evidence for prevention of thermal injuries in children.

**Table 4:** Recommendations and sources of the evidence for preventing thermal injuries

Recommendations	Guidelines and/or institutions recommending the intervention	Source of the evidence
**Smoke alarms** - Have a functional smoke detector alarm - Legislations	- WHO 2011; global burns guide [3] - WHO 2008; European region [1] - PrevInfad 2011 [42] - RCPCH 2019 [2] - CDC 2019 [16] - NICE 2010b [35] - EuroSafe 2006 [13]	- Kendrick 2013; Cochrane systematic review [50] - Kendrick 2012; Cochrane systematic review [45] - Turner 2011; Cochrane systematic review [49] - DiGuiseppi 2001; Cochrane systematic review [59] - Ballesteros 2005, cohort study [60] - Smith 2006; cohort study [61] - Towner 2001, systematic review [62]
**Control of hot water** - Regulating the temperature of water flowing from household taps - Safe pre-set temperature for all water heaters - Legislations - Educational programmes	- WHO 2011; global burns guide [3] - WHO 2008; European region [1] - PrevInfad 2011 [42] - RCPCH 2019 [2] - CDC 2019 [16] - NICE 2010b [35] - EuroSafe 2006 [13]	- Kendrick 2013; Cochrane systematic review [50] - Kendrick 2012; Cochrane systematic review [45] - Kendrick 2011, RCT [63]
**Non-flammable fabrics** - Use of fire-retardant materials for making children’s sleepwear - Legislation	- WHO 2011; global burns guide [3] - WHO 2008; European region [1] - EuroSafe 2006 [13]	- Liao 2000; law and regulations [64]
**Fire sprinkler system**	- WHO 2011; global burns guide [3] - WHO 2008; European region [1]	(*See original documents*)
**Electrical safety**	- WHO 2011; global burns guide [3]	- Kendrick 2012; Cochrane systematic review [45]
**Lighters** - Legislations for child-resistant lighters and self-extinguishing cigarettes - Legislations	- WHO 2011; global burns guide [3] - WHO 2008; European region [1] - EuroSafe 2006 [13]	- European laws and regulations 2006 [65]
**Fireworks** - Legislation banning the manufacture and sale of fireworks - Education campaigns	- WHO 2011; global burns guide [3] - WHO 2008; European region [1] - NICE 2010b [35] - EuroSafe 2006 [13]	(*See original documents*)
**Have an escape plan**	- RCPCH 2019 [2] - CDC 2019 [16]	- Kendrick 2012; Cochrane systematic review [45]
**Possession of a fire extinguisher**		- Kendrick 2012; Cochrane systematic review [45]
**Use of fire guards**	- RCPCH 2019 [2]	- Kendrick 2012; Cochrane systematic review [45]
**Keeping hot drinks/food out of reach of children**	- RCPCH 2019 [2]	- Kendrick 2012; Cochrane systematic review [45]
**Storage of matches or lighters out of reach of children**	- RCPCH 2019 [2]	- Kendrick 2012; Cochrane systematic review [45]

***Abbreviations*:***CDC* Centers for Disease Control and Prevention, *NICE* National Institute for Health and Care Excellence, *PrevInfad* PrevInfad workgroup from the Spanish Association of Primary Care Pediatrics, *RCPCH* Royal College of Paediatrics and Child Health, *WHO* World Health Organization

### Falls

A fall is defined as ‘an event that results in a person coming to rest inadvertently on the ground or floor or other lower level’ [1]. Falls occur frequently and children are especially vulnerable. While most of them are not serious, some can cause serious injury, disability and fatal outcome. In young children, falls are the most common cause of fatal and serious head injuries [1]. Once more, inequalities exist and if all countries matched the lowest rate in the European region, 90% of deaths could be prevented.

One of the three NICE documents published in 2010 on prevention strategies for unintentional strategies recommend some actions to be taken for preventing falls in children [35].

We summarize in Table 5 the recommendations and sources of the evidence for prevention of falls in children.

**Table 5:** Recommendations and sources of the evidence for preventing falls

Recommendations	Guidelines and/or institutions recommending the intervention	Source of the evidence
**Replace or modify unsafe products**	- WHO 2008; European region [1]	(*See original documents*)
**Safety equipment** - Install stair gates - Install windows guards or locks - Possession of non-slip bathmats or decals	- WHO 2008; European region [1] - RCPCH 2019 [2] - CDC 2019 [16] - NICE 2010b [35] - EuroSafe 2006 [13]	- Kendrick 2013; Cochrane systematic review [50] - Kendrick 2012; Cochrane systematic review [45] - Watson 2005; RCT [61] - Towner 2001, systematic review [62]
**Protective equipment** - Use of helmet while riding a horse - Use of helmet, wrist guards, and knee and elbow pads while skating	- WHO 2008; European region [1] - CDC 2019 [16]	(*See original documents*)
**Playground** - Implementation of playground standards (eg. surface material)	- WHO 2008; European region [1] - CDC 2019 [16] - EuroSafe 2006 [13]	- Khambalia 2006, systematic review [66] - Towner 2001, systematic review [62]
**Education** - Implementation of multifaceted community programmes	- WHO 2008; European region [1]	- Towner 2001, systematic review [62] - McClure 2005, systematic review [67]
**Do not use baby walkers** - Legislation banning baby walkers or requiring product modification	- PrevInfad 2011 [42] - RCPCH 2019 [2] - EuroSafe 2006 [13]	- Kendrick 2012; Cochrane systematic review [45] - Khambalia 2006, systematic review [66]
**Supervision** - Supervise at all time around fall hazards	- RCPCH 2019 [2] - CDC 2019 [16]	- Kendrick 2012; Cochrane systematic review [45]

***Abbreviations*:***CDC* Centers for Disease Control and Prevention, *NICE* National Institute for Health and Care Excellence, *PrevInfad* PrevInfad workgroup from the Spanish Association of Primary Care Pediatrics, *RCPCH* Royal College of Paediatrics and Child Health, *WHO* World Health Organization

## Summary of findings

- In 2008, the World Health Organization (WHO) published a comprehensive report with strategies for child injury prevention for the European region. More recently, the WHO published several guidance documents focused on one area such as drowning, usually with a global focus.
- The PrevInfad workgroup (Spanish Association of Primary Care Pediatrics) updated their document on road safety in April 2019, providing recommendations and a summary of the existing evidence. They are currently working on updating the document on home safety, which includes prevention of drowning, poisoning, thermal injuries and falls.
- The key strategies for preventing unintentional injuries are a combination of environmental and behaviour modification, that can be achieved through engineering, enforcement and education. Consequently, for this kind of strategies, it is important to evaluate the effectiveness of both the intervention itself, and the way the intervention is advised to parents and caretakers so that there is good compliance of the recommendation.
- Preventive strategies for injuries in childhood are mainly based on surveillance data and the identification of risk factors.

## Data Availability

Not applicable.

## References

[1] World Health Organization Regional Office for Europe. European report on child injury prevention [Internet]. 2008 [cited 2019 Oct 18]. Available from: https://www.euro.who.int/en/publications/abstracts/european-report-on-child-injury-prevention

[2] Kendrick D. Unintentional injuries and their prevention. In: Emond A, editor. Health for all children. fifth. Oxford: Oxford University Press; 2019:160–80.

[3] Mock C, Peck M, Juillard C, Meddings D, Gielen A, McKenzie L (2011). Burn prevention. Success Stories Lessons Learned.

[4] World Health Organization. Prevention and treatment of HIV and other sexually transmitted infections among men who have sex with men and transgender people. Recommendations for a public health approach. Geneva; 2011 [cited 2019 Oct 18]. Available from: https://www.who.int/hiv/pub/guidelines/msm_guidelines2011/en/26158187

[5] Esparza-Olcina M, PrevInfad Group. Prevención de lesiones infantiles por accidente de tráficos [Internet]. Recomendaciones PrevInfad/PAPPS. 2019 [cited 2019 Oct 10]. Available from: http://previnfad.aepap.org/monografia/accidentes-trafico

[6] World Health Organization. Global status report on road safety [Internet]. Geneva; 2018 [cited 2019 Oct 18]. Available from: https://www.who.int/publications/i/item/9789241565684

[7] United Nations. United Nations Road Safety Collaboration. Publications [Internet]. [cited 2019 Oct 10]. World Health Organization; 2019. Available from: https://www.who.int/roadsafety/publications/en/

[8] World Health Organization. Road traffic injuries publications and resources [Internet]. Violence and Injury Prevention. 2019 [cited 2019 Oct 15]. Available from: https://www.who.int/violence_injury_prevention/publications/road_traffic/en/

[9] Peden M, Oyegbite K, Ozanne-Smith J, Hyder AA, Branche C, Rahman AF, et al. World report on child injury prevention [Internet]. 2008 [cited 2019 Oct 18]. Available from: https://www.who.int/violence_injury_prevention/child/injury/world_report/en/26269872

[10] US Preventive Services Task Force. Motor Vehicle Occupant Restraints: Counseling [Internet]. 2007 [cited 2019 Oct 10]. Available from: https://www.uspreventiveservicestaskforce.org/BrowseRec/ReferredTopic/243

[11] NICE. NICE guidance [Internet]. 2010 [cited 2019 Oct 15]. Available from: https://www.nice.org.uk/search?q=Unintentional+injuries+among+under-15s

[12] EuroSafe. Working together to reduce the burden of home and leisure accidents in Europe [Internet]. 2019 [cited 2019 Oct 16]. Available from: http://www.eurosafe.eu.com/home

[13] EuroSafe. Child Safety Good Practice Guide: Good investments in unintentional child injury prevention and safety promotion [Internet]. 2006 [cited 2019 Oct 18]. Available from: https://www.childsafetyeurope.org/publications/goodpracticeguide/info/good-practice-guide.pdf

[14] World Health Organization. Review of literature on new evidence on road traffic injury risk factors and interventions [Internet]. 2019 [cited 2019 Oct 15]. Available from: https://www.who.int/violence_injury_prevention/road_traffic/RS_Roard_Factors_RFP.pdf?ua=1

[15] World Health Organization, FIA Foundation for the Automobile and Society. Seat-belts and child restraints: a road safety manual for decision-makers and practitioners. London; 2009 [cited 2019 Oct 18]. Available from: https://www.who.int/roadsafety/projects/manuals/seatbelt/en/

[16] Centers for Disease Control and Prevention. Protect the Ones You Love: Child Injuries are Preventable [Internet]. 2019 [cited 2019 Oct 10]. Available from: https://www.cdc.gov/safechild/index.html

[17] Arbogast K, Jermakian J, Kallan M, Durbin D (2009). Effectiveness of belt positioning booster seats: an updated assessment. Pediatrics..

[18] Zaloshnja E, Miller T, Hendrie D (2007). GradDip (Road Safety). Effectiveness of child safety seats vs safety belts for children aged 2 to 3 years. Arch Pediatr Adolesc Med.

[19] Elliott MR, Kallan MJ, Durbin DR, Winston FK (2006). Effectiveness of child safety seats vs seat belts in reducing risk for death in children in passenger vehicle crashes. Arch Pediatr Adolesc Med.

[20] Sauber-Schatz E, Thomas A, Cook L (2015). Motor vehicle crashes, medical outcomes, and hospital charges among children aged 1–12 years — Crash outcome data evaluation system, 11 States, 2005–2008. Centers Dis Control Prev Morb Mortal Wkly Rep.

[21] MAPFRE. Estudios de sillas para los niños en coches: Mirando hacia atrás [Internet]. 2011 [cited 2019 Oct 10]. p. 1–16. Available from: https://sillasdecoche.fundacionmapfre.org/ infantiles/images/segvial-mirando-hacia-atras_tcm725–93539.pdf.

[22] Mcmurry TL, Arbogast KB, Sherwood CP, Vaca F, Bull M, Crandall JR (2018). Rear-facing versus forward-facing child restraints: an updated assessment. BMJ..

[23] Jakobsson L, Isaksson-Hellman I. Safety for the growing child: experiences from Swedish accident data [Internet]. 19th international technical conference on the enhanced safety of vehicles. Washington DC; 2005. [cited 2019 Jul 13]. Available from: https://trid.trb.org/view/815353.

[24] Gloyns P, Roberts J. An Accident Study of the Performance of Restraints Used by Children Aged Three Years and Under [Internet]. Brussels; 2008 [cited 2019 Oct 18]. Available from: https://www.anec.eu/attachments/ANEC-R&T-2008-TRAF-003.pdf

[25] Lennon A, Siskind V, Haworth N (2008). Rear seat safer: seating position, restraint use and injuries in children in traffic crashes in Victoria, Australia. Accid Anal Prev.

[26] Kallan MJ, Durbin DR, Arbogast KB (2008). Seating patterns and corresponding risk of injury among 0- to 3-year-old children in child safety seats. Pediatrics..

[27] Ehiri J, Ejere H, Magnussen L, Emusu D, King W, Osberg S. Interventions for promoting booster seat use in four to eight year olds travelling in motor vehicles (Review). Cochrane Database Syst Rev. 2006:CD004334. 10.1002/14651858.CD004334.pub2 PMC8805601 16437484

[28] World Health Organization. Helmets: a road safety manual for decision-makers and practitioners. Geneva: World Health Organization; 2006. [cited 2019 Oct 19]. Available from: https://www.who.int/publications/i/item/helmets-a-road-safety-manual-for-decision-makers-and-practitioners.

[29] US Community Preventive Services Task Force. Motor vehicle - Related injury prevention: use of motorcycle helmets, universal helmet laws. Task Force finding and rationale statement [Internet]. The Community Guide. 2013 [cited 2019 Oct 19]. Available from: https://www.thecommunityguide.org/findings/motor-vehicle-injury-motorcycle-helmets-universal-helmet-laws

[30] Owen R, Kendrick D, Mulvaney C, Coleman T, Royal S. Non-legislative interventions for the promotion of cycle helmet wearing by children (Review). Cochrane Database Syst Rev. 2011:CD003985. 10.1002/14651858.CD003985.pub3 PMC7390328 22071810

[31] Liu B, Ivers R, Norton R, Boufous S, Blows S, Lo S. Helmets for preventing injury in motorcycle riders (Review). Cochrane Database Syst Rev. 2008:CD004333. 10.1002/14651858.CD004333.pub3 PMC13092890 18254047

[32] Macpherson A, Spinks A. Bicycle helmet legislation for the uptake of helmet use and prevention of head injuries (Review). Cochrane Database Syst Rev. 2008:CD005401. 10.1002/14651858.CD005401.pub3 PMC6823199 18646128

[33] Thompson D, Rivara F, Thompson R. Helmets for preventing head and facial injuries in bicyclists (Review). Cochrane Database Syst Rev. 1999:CD001855. 10.1002/14651858.CD001855 PMC7025438 10796827

[34] World Health Organization. Pedestrian safety. A road safety manual for decision-mkers and practitioners [Internet]. United Nations Road Safety Collaboration. 2013 [cited 2019 Oct 10]. Available from: https://www.who.int/roadsafety/projects/manuals/pedestrian/en/

[35] NICE. Unintentional injuries on the road: interventions for under 15s. 2010.

[36] Kwan I, Mapstone J. Interventions for increasing pedestrian and cyclist visibility for the prevention of death and injuries (Review). Cochrane Database Syst Rev. 2009:CD003438. 10.1002/14651858.CD003438.pub2 PMC8713592 17054171

[37] Duperrex O, Bunn F, Roberts I (2002). Safety education of pedestrians for injury prevention: a systematic review of randomised controlled trials. BMJ..

[38] Duperrex O, Roberts I, Bunn F. Safety education of pedestrians for injury prevention (Review). Cochrane Database Syst Rev. 2002:CD001531. 10.1002/14651858.CD001531 PMC7025789 12076415

[39] Turner C, McClure R, Nixon J, Spinks A (2004). Community ­ based programmes to prevent pedestrian injuries in children 0-14 years: a systematic review. Inj Control Saf Promot.

[40] Bunn F, Collier T, Frost C, Ker K, Steinbach R, Roberts I, et al. Area-wide traffic calming for preventing traffic related injuries (Review). Cochrane Database Syst Rev. 2003:CD003110. 10.1002/14651858.CD003110 PMC8693445 12535454

[41] World Health Organization. Preventing drowning: an implementation guide. Geneva: World Health Organization; 2017. Licence: CC BYNC-SA 3.0 IGO [cited 2019 Oct 19]. Available from: https://www.who.int/publications/i/item/preventing-drowning-an-implementation-guide.

[42] Esparza-Olcina M, PrevInfad Group. Prevención de lesiones infantiles por accidente doméstico. Recomend PrevInfad/PAPPS. 2011. p. 1–30. Available from: http://previnfad.aepap.org/monografia/accidentes-domesticos [cited 2019 16 Oct]

[43] Thompson D, Rivara F. Pool fencing for preventing drowning of children (Review). Cochrane Database Syst Rev. 1998:CD001047. 10.1002/14651858.CD001047 PMC8407364 10796742

[44] Neto C, Barreiros J, Vieira F, Cordovil R, Aparício P, Cardoso de Menezes H. Dimensions and design of swimming pool fences and balcony and stairs barriers to protect children from falling and from passing trough, bellow or above. Brussels; 2008.

[45] Kendrick D, Young B, Mason-Jones A, Ilyas N, Achana F, Cooper N, et al. Home safety education and provision of safety equipment for injury prevention (Review). Cochrane Database Syst Rev. 2012:CD005014. 10.1002/14651858.CD005014.pub3 PMC9758703 22972081

[46] Santé Publique France. Surveillance épidémiologique des noyades. Enquête NOYADES 2015. 2015.

[47] World Health Organization. Poisoning prevention and management [Internet]. 2019. [cited 2019 Oct 17]. Available from: https://www.who.int/ipcs/poisons/en/

[48] World Health Organization. The IPCS INTOX Programme [Internet]. International Programme on Chemical Safety. [cited 2019 Oct 17]. Available from: https://www.who.int/ipcs/poisons/intox/en/

[49] Turner S, Arthur G, Lyons R, Weightman A, Mann M, Jones S, et al. Modification of the home environment for the reduction of injuries (Review). Cochrane Database Syst Rev. 2011:CD003600. 10.1002/14651858.CD003600.pub3 PMC7003565 21328262

[50] Kendrick D, Mulvaney C, Ye L, Stevens T, Mytton J, Stewart-Brown S. Parenting interventions for the prevention of unintentional injuries in childhood (Review). Cochrane Database Syst Rev. 2013:CD006020. 10.1002/14651858.CD006020.pub3 PMC8908963 23543542

[51] Flanagan R, Rooney C, Griffiths C (2005). Fatal poisoning in childhood, England & Wales 1968-2000. Forensic Sci Int.

[52] Roberts DM, Karunarathna A, Buckley NA, Manuweera G, Sheriff MHR, Eddleston M (2003). Influence of pesticide regulation on acute poisoning deaths in Sri Lanka. Bull World Health Organ.

[53] EUR-Lex. Directive 1999/45/EC of the European Parliament and of the Council of 31 May 1999 concerning the approximation of the laws, regulations and administrative provisions of the Member States relating to the classification, packaging and labelling of dangerous [Internet]. 2008 [cited 2019 Oct 17]. Available from: https://eur-lex.europa.eu/legal-content/EN/TXT/?uri=CELEX%3A31999L0045

[54] World Health Organization. Poisons centres [Internet]. International Programme on Chemical Safety. [cited 2019 Oct 17]. 2019. Available from: https://www.who.int/ipcs/poisons/centre/en/

[55] Bateman DN, Good AM, Kelly CA, Laing WJ (2002). Web based information on clinical toxicology for the United Kingdom: uptake and utilization of TOXBASE in 2000. J Clin Pharmacol.

[56] Höjer J, Troutman W, Hoppu K, Erdman A, Benson B, Mégarbane B (2013). Position paper update: ipecac syrup for gastrointestinal decontamination. Clin Toxicol.

[57] Mock C, Peck M, Peden M, Krug E. A WHO plan for burn prevention and care [Internet]. Geneva: World Health Organization; 2008. [cited 2019 Oct 21]. Available from: https://apps.who.int/iris/handle/10665/97852.

[58] Turner C, Spinks A, McClure R, Nixon J. Community-based interventions for the prevention of burns and scalds in children (Review). Cochrane Database Syst Rev. 2012:CD004335. 10.1002/14651858.CD004335.pub2 PMC6464795 15266531

[59] DiGuiseppi C, Goss C, Higgins J. Interventions for promoting smoke alarm ownership and function (Review). Cochrane Database Syst Rev. 2010:CD002246. 10.1002/14651858.CD002246 PMC7017850 11406039

[60] Ballesteros M, Jackson M, Martin M (2005). Working toward the elimination of residential fire deaths: the Centers for Disease Control and Prevention’s smoke alarm installation and fire safety education (SAIFE) program. J Burn Care Rehabil.

[61] Smith G, Splaingard M, Hayes J, Xiang H (2019). Comparison of a personalized parent voice smoke alarm with a conventional residential tone smoke alarm for awakening children. Pediatrics..

[62] Towner E, Dowswell T, Jarvis S (2001). Updating the evidence. A systematic review of what works in preventing childhood unintentional injuries: part 2. Inj Prev.

[63] Kendrick D, Stewart J, Smith S, Coupland C, Hopkins N, Groom L (2011). Randomised controlled trial of thermostatic mixer valves in reducing bath hot tap water temperature in families with young children in social housing. Arch Dis Child.

[64] Liao C, Rossignol A (2000). Landmarks in burn prevention. Burns..

[65] EUR-Lex. Decision 2006/502/EC Commission Decision of 11 May 2006 requiring Member States to take measures to ensure that only lighters which are child-resistant are placed on the market and to prohibit the placing on the market of novelty lighters. Brussels, Office of the Official Publications of the European Communities. 2006 [cited 2019 Oct 18]. Available from: https://eur-lex.europa.eu/legal-content/EN/ALL/?uri=CELEX%3A32006D0502

[66] Khambalia A, Joshi P, Brussoni M, Raina P, Morrongiello B, Macarthur C (2006). Risk factors for unintentional injuries due to falls in children aged 0–6 years: a systematic review. Inj Prev..

[67] McClure R, Nixon J, Spinks A, Turner C (2005). Community-based programmes to prevent falls in children: a systematic review. J Pediatr Child Heal.

