# Correction to: Prevention of unintentional injuries in children under five years

**DOI:** 10.1186/s12887-021-02908-5

**Published:** 2021-10-06

**Authors:** Sophie Jullien

**Affiliations:** Barcelona Institute for Global Health, University of Barcelona, Barcelona, Spain

**Correction to: BMC Pediatr 21, 311 (2021)**

**https://doi.org/10.1186/s12887-021-02517-2**

After publication of this supplement article [1], it was brought to our attention that the article had published with a couple of errors in the reference list: firstly, references 35 and 43 had been combined into an incorrect version of reference 35; secondly, reference 67 has been erroneously omitted.

The references have since been corrected in the original article and may be found detailed below:

[35] NICE. Unintentional injuries on the road: interventions for under 15s [Internet]. 2010 [cited 2019 Oct 21]. p. 1–46. Available from: https://www.nice.org.uk/guidance/ph31

[43] NICE. Unintentional injuries: prevention strategies for under 15s [Internet]. 2010 [cited 2019 Oct 21]. p. 1–88. Available from: https://www.nice.org.uk/guidance/ph29

[67] Watson M, Kendrick D, Coupland C, Woods A, Futers D, Robinson J. Providing child safety equipment to prevent injuries: randomised controlled trial. BMJ. 2005;330:178–82.

The author apologizes for any inconvenience caused.
